# Risk Factors of Atrial Fibrillation in Patients with Heart Failure

**DOI:** 10.7759/cureus.3774

**Published:** 2018-12-24

**Authors:** Alam Sarfraz, Parveen Akhtar, Syed N Hassan Rizvi, Musa Karim

**Affiliations:** 1 Cardiology, National Institute of Cardiovascular Diseases, Karachi, PAK; 2 Miscellaneous, National Institute of Cardiovascular Diseases, Karachi, PAK

**Keywords:** atrial fibrillation, risk factors, heart failure

## Abstract

Introduction

Coexistence of atrial fibrillation (AF) in patients with heart failure (HF) is a common phenomenon associated with poor prognosis. Therefore, this study was designed with an aim to estimate the different risk factors of atrial fibrillation (AF) in patients with HF.

Methods

In this study, patients of either gender, 18 to 80 years of age, and with echocardiographic confirmation of HF presenting at the adult cardiology department of the National Institute of Cardiovascular Diseases (NICVD), Karachi, Pakistan were consecutively included. Patients diagnosed with chronic obstructive airway diseases, pneumonia, or pericarditis, and patients diagnosed with existing AF were excluded from the study. Data regarding demographic and clinical risk factors of AF were obtained using a structural proforma.

Results

Out of 150 patients, 59.3% (89) were females, and the mean age was 50 ± 16 years. A majority of the patients, 55.3% (83), had a history of rheumatic heart diseases (RHD) and 22.7 (34) percent had a history of transient ischemic attack (TIA) or cerebrovascular accident (CVA). On echocardiography, 28.0% (42) of the patients had right ventricular (RV) dysfunction, and the clot was seen in 28.0% (42) of the patients. Mitral stenosis (MS) and mitral regurgitation (MR) were observed in 34.5% (61) and 29.3% (52) of the patients, respectively.

Conclusion

We observed that the adult population with HF tends to have multiple risk factors of AF. More coordinated efforts are needed by the healthcare professionals to understand and manage these coupled conditions.

## Introduction

Cardiovascular diseases (CVD) account for nearly 10% of the worldwide burden of diseases, measured by a combination of death and disability, and this is expected to rise to almost 15% by 2020 [1]. Heart failure (HF) is the most commonly observed manifestation of the acute coronary syndrome (ACS) and is related to increased adverse outcomes [2-3]. HF accounts for more than a 100 billion dollars of a direct and indirect global economic burden. Middle- and low-income countries bear the major share of indirect cost [4]. Coexistence of atrial fibrillation (AF) and HF is a common phenomenon, with both worsening the course of one another. The presence of both the conditions in a patient is associated with poor prognosis [5]. A complex interplay exists between HF and AF, and the development of AF is influenced by HF, while the stimulation of HF is influenced by AF. HF with both reduced and preserved ejection fraction (EF) is worsened by the preexisting AF. The individual clinical factors known to increase a patient’s susceptibility include advanced age, diabetes mellitus (DM), and congestive heart failure (CHF) [6].

The prevalence of AF in the European population is around 1% to 2%; however, it has been increasingly observed in adultery population [7]. Depending on the age of the patients and the type of HF, the prevalence rate of AF varies from 13% to 41% [4,8]. A study conducted on 10,701 patients with HF, admitted to the hospital in 24 countries in Europe, demonstrated that only 9% had new-onset AF, while 34% had preexisting AF [9].

Clinical risk factors for the development of AF in the general population have been characterized by different risk factors like age, race, body weight, and height, and blood pressure, antihypertensive medication, diabetes, and a history of myocardial infarction (MI), and CVDs [10-12]. In addition to structural heart diseases, a number of other factors may contribute to the development of AF including inflammation and autonomic. The prognostic impact of AF in patients with HF is well established. A number of studies have reported a deleterious effect on outcomes such as increased risk of mortality, stroke, and morbidity [13].

The frequency and risk factors of AF in the Pakistani population are not known. Therefore, this study was designed with the rationale to estimate the different risk factors of AF in patients with HF.

## Materials and methods

This cross-sectional study was performed at the adult cardiology department of the National Institute of Cardiovascular Diseases (NICVD), Karachi, Pakistan from February 2017 to July 2017. This study was approved by the ethical review committee (ERC) of the institution (ERC approval number: ERC-07/2017). In this study, patients of either gender above 18 years of age with echocardiographic confirmation of HF were consecutively included. Informed consent regarding participation in this study was obtained from all the enrolled patients. Exclusion criteria for the study were patients below 18 years or above 80 years of age, patients diagnosed with chronic obstructive airway diseases, pneumonia, or pericarditis, and patients diagnosed with existing AF. AF was diagnosed based on electrocardiographic (ECG) characteristics including irregular R-R intervals (when atrioventricular conduction is present), an absence of distinct repeating P waves, and irregular atrial activity. The risk factors of AF were categorized as demographic characteristics (advanced age and obesity), clinical manifestations (blood pressure, heart rate, shortness of breath, angina, dizziness, palpitation, and orthopnea), clinical history (hypertension (HTN), dyslipidemia, DM, and history of transient ischemic attack [TIA], ischemic heart diseases [IHD], cerebrovascular accident [CVA], MI, and rheumatic heart diseases [RHD]), ECG changes, and echocardiography findings (the presence of clot, valvular lesions, right ventricular [RV] dysfunction, left atrial size, and pulmonary arterial pressure [PAP]). ECG and echocardiographic findings were analyzed independently by three cardiologists and the agreement of any two regarding a certain finding was considered as the final finding. All the collected information was recorded on a pre-defined structural proforma.

After a thorough literature review, no data were available regarding the risk factors of AF in patients with HF for the Pakistani population; therefore, the sample size for the study was calculated at 95% confidence level, with 50% prevalence guess, and 8% margin of error, and based on these assumptions, the minimum achievable sample size for the study was 151.

Collected data were entered and evaluated for data entry and collection errors, and data analysis was carried out using IBM SPSS Statistics for Windows, Version 21.0. (IBM Corp., Armonk, NY, US). No imputation was performed for the variables with missing data; rather such cases were excluded list-wise from the analysis. Under the assumption of normality of the distribution, quantitative (continuous) information was summarized by calculating the mean ± standard deviation (SD), while for the categorical variables, the frequency and percentage were computed.

## Results

A total of 150 patients were included in this study: 59.3% (89) were females, and the mean age was 50 ± 16 years. A majority, 94.0% (141), of the patients were married and 19.3% (29) were above 65 years of age. A majority of the patients, 55.3% (83), had a history of RHDs, 22.7% (34) had a history of TIAs or CVAs. Majority of the patients, 89.3% (134), presented with SOB class IV. Demographic characteristics, clinical manifestations, and clinical history of the patients are presented in Table 1.

**Table 1:** Demographic characteristics, clinical manifestations, and clinical history

Characteristics	Percentage (*n*)
Gender distribution	
Female	59.3 (89)
Male	40.7 (61)
Marital Status	
Married	94.0 (141)
Unmarried	6.0 (9)
Age distribution	
18 to 40 years	30.7 (46)
41 to 65 years	50.0 (75)
More than 65 years	19.3 (29)
Clinical history	
Hypertension	42.7 (64)
Diabetes	6.7 (10)
Smoker	8.0 (12)
Ischemic heart disease (IHD)	2.7 (4)
Myocardial infarction (MI)	5.3 (8)
Rheumatic heart diseases (RHD)	55.3 (83)
Transient ischemic attack (TIA) / Cerebrovascular accident (CVA)	22.7 (34)
Clinical manifestation	
Systolic blood pressure (SBP)	115.5 ± 21.5 mmHg
Pulse rate	123.6 ± 29.6 bpm
Serum creatinine	1.1 ± 0.6 mg/dL
Hemoglobin (HB)	11.7 ± 1.9 gm/dL
Shortness of breath (SOB - IV)	89.3 (134)
Angina	5.3 (8)
Dizziness	35.3 (53)
Palpitation	78.7 (118)
Orthopnea	46.0 (69)

Radiological cardiomegaly was observed in 66.0% (99) of the patients, left ventricular hypertrophy (LVH) on ECG was observed for a majority, 40.0% (60), of the patients, and 31.3% (47) of the ECGs were normal. In 28.0% (42) of the patients, clots were on observed on ECG, and RV dysfunction was observed in 28.0% (42) of the patients. The major valvular lesions were mitral stenosis (MS) and mitral regurgitation (MR), which were observed in 34.5% (61) and 29.3% (52) of the patients, respectively. ECG changes and echocardiography findings are presented in Table 2.

**Table 2:** ECG changes and echocardiography findings ECG: electrocardiography

Characteristics	Percentage (*n*)
ECG Changes	
Ischemic Changes	22.0 (33)
Left ventricular hypertrophy (LVH)	40.0 (60)
Left bundle branch block (LBBB)/Right bundle branch block (RBBB)	6.7 (10)
Normal	31.3 (47)
Echocardiography findings	
Right ventricular (RV) dysfunction	29.3 (44)
Presence of Clot	28.0 (42)
Valvular Lesion	
Mitral stenosis (MS)	34.5 (61)
Tricuspid regurgitation (TR)	19.8 (35)
Mitral regurgitation (MR)	29.3 (52)
Aortic regurgitation (AR)	1.7 (3)
Aortic stenosis (AS)	1.2 (2)
Other Lesions	13.5 (24)
Left atrial (LA) size	46.7 ± 14.0 mm
Pulmonary arterial pressure (PAP)	39.4 ± 13.8 mmHg

## Discussion

The study of risk factors of AF in adult individuals is important in the current era of preventive cardiology. This study was conducted with the aim of estimating the burden of the risk factors of AF in patients presenting with HF. Studies conducted in the past have identified various clinical risk factors such as advanced age, body mass index (BMI), HTN, DM, and past history of MI [10-12]. Although it is not well established whether HF induces AF or vice versa; however, the pre-existence of AF in patients with HF is an independent prognostic marker.

In our study, the mean age of the patients was 50 ± 16 years, ranging between 19-75 years, and a majority, 50.0% (75), of the patients belong to the age groups of 41 to 65 years. Only 19.3% (29) patients were above 65 years of age. We observed an intense risk profile of AF in our study sample with hypertension in 42.7%, history of RHDs in 55.3%, and a history of TIA/CVA in 22.7% of the patients. RV dysfunction was observed in 29.3%, and echocardiographic assessment showed the presence of a clot in 28.0% of the patients. Among other risk factors, MS was observed in 34.5% of the patients and MR was found in 29.3% of the patients. Clinical history of RHDs and HTN are the primary determinants of AF and other ceaseless cardiovascular sicknesses as reported in numerous studies [13-15].

Most of the patients in our study were females (59.3%) which is comparable to the observations done by other studies [16-17]. A family history of IHDs was found in 4% of our patients, which is much less than 17.3% reported in a study conducted in London by Khazanie et al. [16]. A history of MI was present in 5.3% and 8% of the patients were smokers. Finally, in our study, we observed that adult population with HF tends to have multiple risk factors for the development of AF. Meticulous counseling and management strategies need to be put forth for this population to minimize the prognostic impact of AF in patients with HF. Even with this information, many questions regarding adult patients with HF still exist, for instance, no data are available regarding follow-up, and the effect of treatment strategies on morbidity and mortality has not been definitively reported for our population in these patient groups. In order to answer these questions, prospective studies with multivariate analysis of risk factors are recommended. First-degree relatives of patients with HF should be educated and screened for the presence of traditional risk factors of AF.

## Conclusions

We observed that the adult population with HF tends to have multiple risk factors of AF. Therefore, more coordinated efforts are needed by healthcare professionals to understand and manage these coupled conditions.   
